# Evaluation of the first annular pulley stretch effect under isometric contraction of the flexor tendon in healthy volunteers and trigger finger patients using ultrasonography

**DOI:** 10.1186/s12891-021-04299-1

**Published:** 2021-05-06

**Authors:** Shinya Tanaka, Kosuke Uehara, Ryota Sugimura, Toshiki Miura, Takashi Ohe, Sakae Tanaka, Yutaka Morizaki

**Affiliations:** 1 Department of Orthopaedic Surgery, The University of Tokyo Hospital, 7-3-1, Hongo, Bunkyo-ku, Tokyo, 113-8655 Japan; 2 Department of Orthopaedic Surgery, JR General Hospital, Tokyo, Japan; 3 Department of Orthopaedic Surgery, NTT Medical Center Tokyo, Tokyo, Japan

**Keywords:** A1 pulley, Flexor tendon, Contracture, Trigger finger, Ultrasonograpghy, Physiotherapy, Stretch, A1 pulley stretch, Stenosing flexor tenosynovitis

## Abstract

**Background:**

Isometric exercises for a flexed finger have been reported to be effective for treating trigger finger as the flexor tendon widens the space under the first annular (A1) pulley towards the palmar destination during the exercise. This study aimed to evaluate the structural changes during the A1 pulley stretch in healthy volunteers and patients with trigger finger using ultrasonography.

**Methods:**

We enrolled 25 male and 14 female patients (39 middle fingers). The thickness of the subcutaneous tissue (parameter a), A1 pulley (parameter b), and the flexor tendon (parameter c) and the distance between the dorsal surface of the flexor tendon and the palmar surface of the metacarpal head (parameter d) were measured using ultrasonography of the metacarpophalangeal joint of the middle finger flexed at 45° at rest (pattern A) and under isometric contraction of the flexor tendon against an extension force of the proximal interphalangeal joint (pattern B).

**Results:**

The average differences between patterns A and B in the healthy volunteers were 0.29 mm (parameter a; *P* = 0.02), 0.017 mm (parameter b; *P* = 0.63), 0.16 (parameter c; *P* = 0.26), and 0.41 (parameter d; *P* = 0.004), and those in patients with trigger finger were 0.22 mm (parameter a; *P* = 0.23), 0.019 mm (parameter b; *P* = 0.85), 0.03 mm (parameter c; *P* = 0.82), and 0.78 mm (parameter d; *P* < 0.001). The distance between the dorsal side of the A1 pulley and the palmar surface of the metacarpal head was also significantly increased by 0.57 mm (8.2%) in healthy volunteers (*P* < 0.001) and 0.81 mm (11%) in patients with trigger finger (*P* < 0.001).

**Conclusions:**

In this study, the space under the A1 pulley was expanded under isometric contraction of the flexor tendon. These findings support the effectiveness of pulley stretch exercises for the trigger finger condition.

## Introduction

Trigger finger is one of the most common diseases that can lead to long-term pain, deformity, and disability [1–4], in which the first annular (A1) pulley becomes stiff and thick [5]. Similarly, the tendon accompanied by synovitis [6] also becomes thick [7], which results in the A1 pulley lumen (A1PL) becoming relatively narrow for the tendon. The management of trigger finger includes both nonsurgical and surgical methods. Non-surgical treatments include the adjustment and modification of activities [8, 9], the use of orthoses and splints [10], and administration of hyaluronic acid [11] and steroid injections [12, 13]. Surgical treatment is performed either as an open or percutaneous procedure [8, 14, 15]. Orthoses and splints are associated with a reduction in the stage of stenosing tenosynovitis, the numeric pain rating scale, and the number of triggering events [10, 16]; however, they are not sufficient for complete recovery. Some drawbacks of steroid injections include pain during the procedure and the risk of tendon rupture [17]. The recurrence rate in the operated trigger finger has been reported to be as low as 0.5% [18]; however, patients may temporarily experience pain on the surgical site postoperatively [3].

As the A1PL becomes relatively narrow for the tendon with triggering, stretching the A1 pulley with an isometric contraction can be effective. Upon flexion of the metacarpophalangeal (MP) joint, the active flexor tendon contraction force and counteracting flexor tendon force can generate contact forces that expand the A1 pulley toward the palm. It has been reported that a combination of this “A1 pulley stretch” and the conventional passive extension stretch of the finger for 30 s, 10 times daily for a month, improved pain and triggering during motion in patients with trigger finger [19]. A cadaveric study reported that A1PLs expanded upon loading of the flexor tendon with the proximal interphalangeal (PIP) and MP joint fixed at a flexion angle [19]. This cadaveric study did not include patients with trigger finger, who may have a stiffer and thicker A1 pulley, thereby making it difficult to expand the A1PL [5]. To the best of our knowledge, no study has proven whether the A1 ligamentous pulley lumen is expanded in healthy volunteers and patients with trigger finger in vivo. We hypothesized that the A1 pulley stretch expands the A1PL in both healthy volunteers and patients with trigger finger. In this study, we evaluated the structural changes around the A1 pulley under isometric contraction of the flexor tendon at 45° flexion of the middle finger at rest (pattern A) and under isometric contraction of the flexor tendon resisted against the force toward the proximal interphalangeal joint extension (pattern B) in healthy volunteers and patients with trigger finger using ultrasonography.

## Patients and methods

**Fig. 1:**
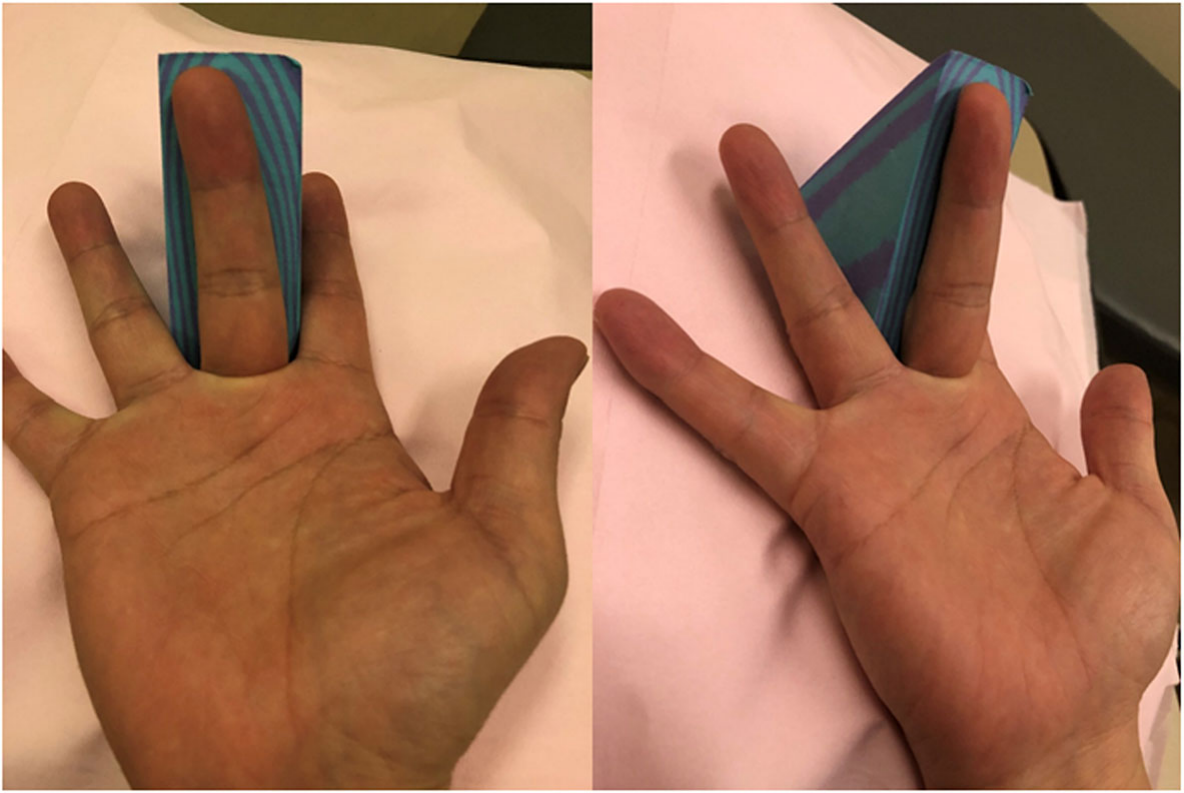
The block, which had a right-angled triangular prism shape and was made of ethylene-vinyl acetate copolymer resin, was used to maintain the finger position throughout the exercise. This fixation block was placed under the base of the proximal phalangeal bone of the middle finger, and the metacarpophalangeal joint was flexed at 45°

**Fig. 2:**
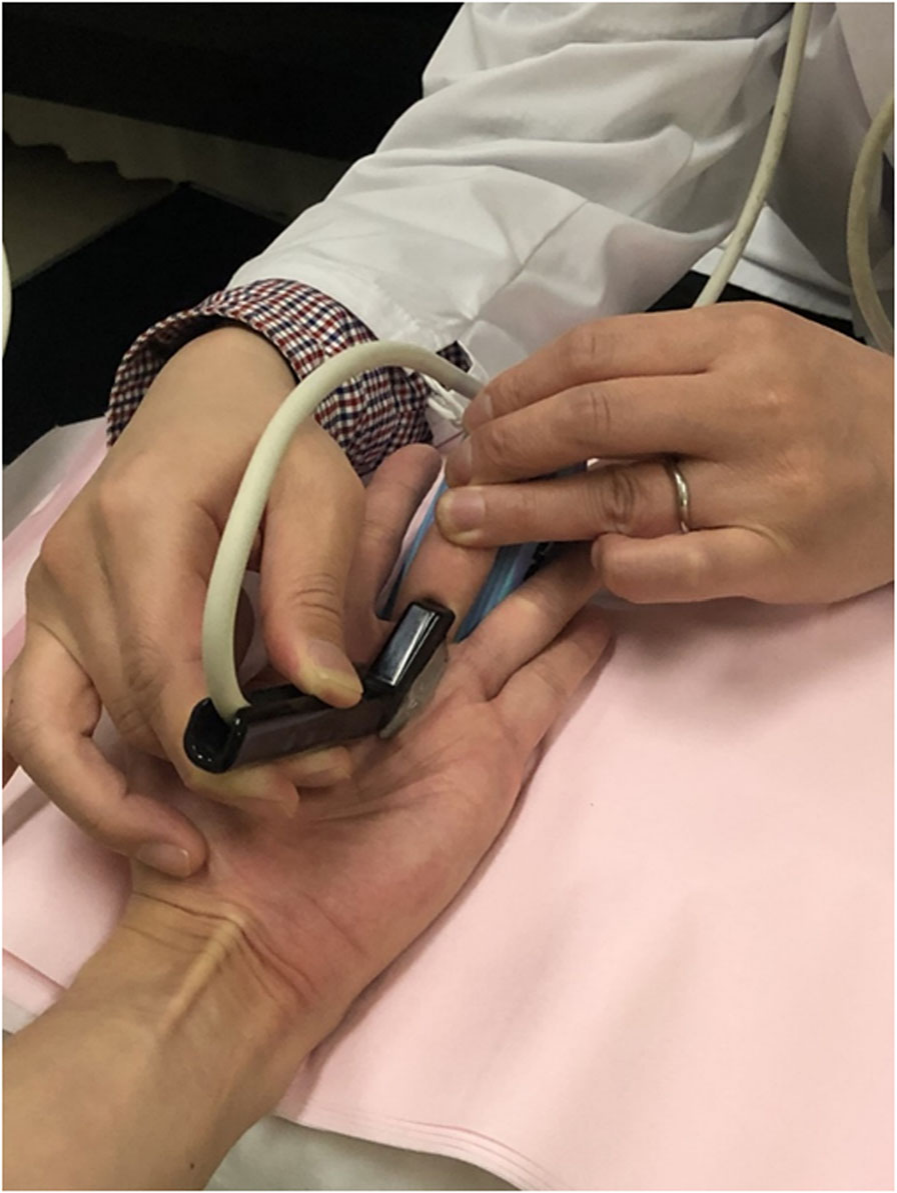
A hockey stick type of probe was used in this study. The examiner applied an ultrasonic probe onto the first annular (A1) tendon sheath and instructed the participants to perform active flexion of the proximal interphalangeal (PIP) joint of the middle finger isometrically to resist the passive extension load for the PIP joint applied by the examiner while keeping the metacarpophalangeal joint flexed at 45° with a custom-made fixing block

**Fig. 3:**
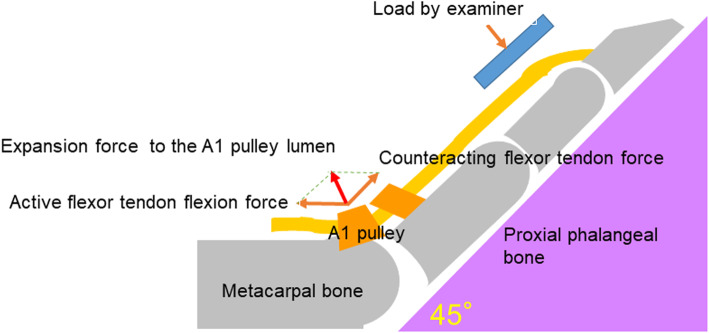
Schema of pattern B with the metacarpophalangeal joint of the middle finger flexed at 45° under isometric contraction of the flexor tendon. The examiner pushes the middle finger toward the dorsal side during the examination. An active flexor tendon contraction force and a counteracting flexor tendon force can generate contact force that expands the first annular (A1) pulley toward the palm side

**Fig. 4:**
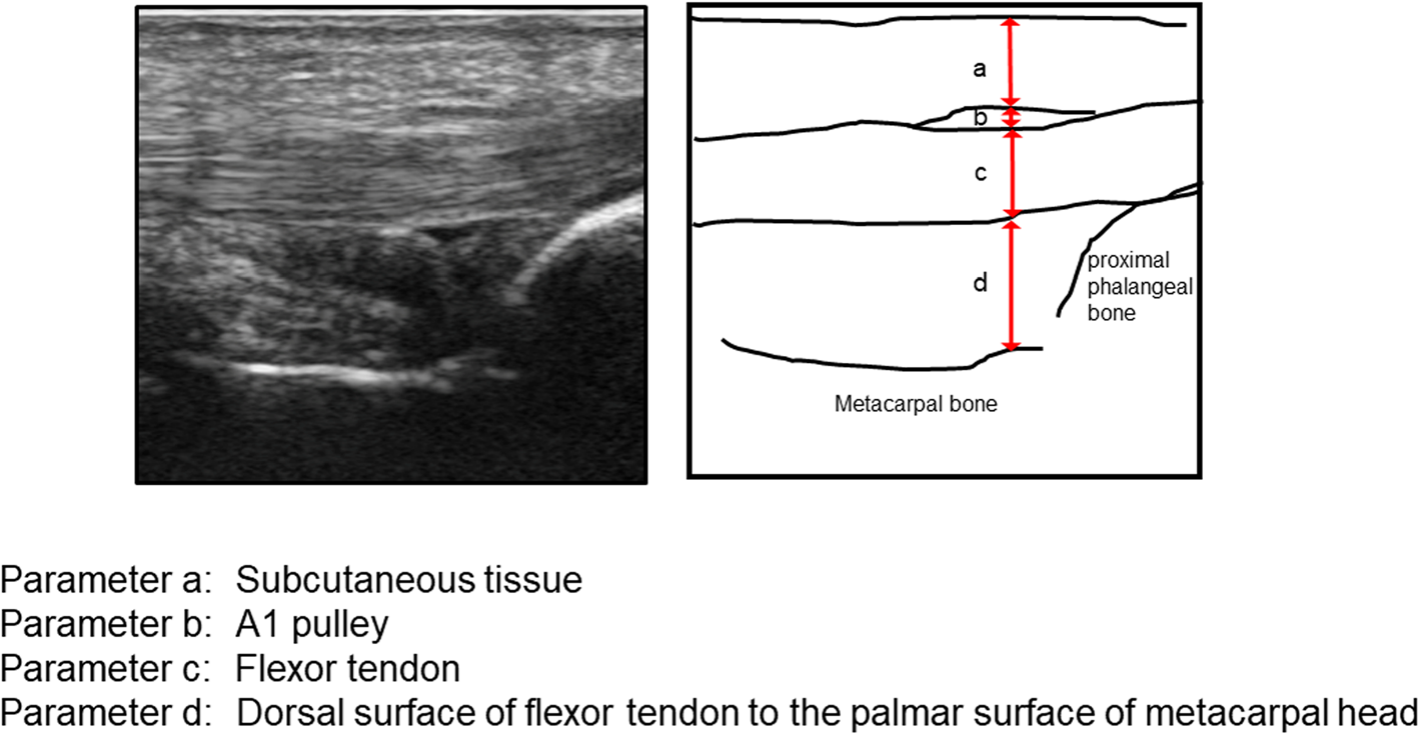
Measured parameters during ultrasonographic examination on ultrasonographic image and schema. The thickness of the (parameter a) subcutaneous tissue, (parameter b) first annular (A1) pulley, (parameter c) flexor tendon, and (parameter d) dorsal surface of the flexor tendon to the palmar surface of the metacarpal head are measured using ultrasonography

This study was conducted with the approval of the Ethics Committee at our institute (reference number: 11627) and abides by the 1964 Helsinki Declaration and its later amendments or comparable ethical standards. Written informed consent was obtained from all the participants. Patient background information collected from the electronic charts included disease duration, number of affected fingers, and presence or absence of diabetes. A Quinnell grading system for trigger finger was used to assess the severity of the disease [20]. Only the middle finger was tested in this study. While testing using ultrasonography, the examinee was seated in front of the table facing the examiner, with the elbow flexed at 90° and the forearm supinated. An ultrasonographic device (HI VISION Avius, Hitachi Medical Corporation, Tokyo, Japan) and a 10 MHz hockey stick-type probe (EUP-054 J, Hitachi Medical Corporation, Tokyo, Japan) were used for this study (Fig. 1). Images were recorded using a long-axis view of the middle finger. The measured parameters are described below. While holding the MP joint flexed at 45° with a custom-made fixing block (pattern A), the examiner applied an ultrasonic probe onto the A1 tendon sheath and measured the thickness of the (parameter a) subcutaneous tissue, (parameter b) A1 pulley, (parameter c) flexor tendon, and (parameter d) dorsal surface of the flexor tendon to the palmar surface of the metacarpal head using a digital caliper. To retain sufficient reproducibility, measurements were performed at the thickest point of the A1 pulley to measure parameter b, and the most volar level of the metacarpal head for the other parameters a, c, and d. Subsequently, the examiner instructed the participants to perform active flexion of the PIP joint of the middle finger with the MP joint flexed at 45° to resist the passive extension load applied by the examiner on the PIP joint, thereby loading an isometric contraction of the finger flexor (pattern B) (Fig. 2). The images of the long-axis view of the middle finger during testing were recorded, and parameters a, b, c, and d were measured digitally (Figs. 3 and 4). The average distances between the dorsal surface of the A1 pulley and the palmar surface of the metacarpal head (parameter c + d), which indicated the diameter of the A1PL in patterns A and B, were compared in healthy volunteers and patients with trigger finger.

We analyzed the relationship between each measurement parameter (a–d) and the patient’s background, disease duration, sex, Quinnell grade, number of affected fingers, and the presence or absence of diabetes.

The resolution of the ultrasonography with a 10 MHz probe used in this study was 0.2 mm. We evaluated intra- and inter-examiner reliability for parameter b in 10 patients, which was 0.90 (95% confidence interval [CI] 0.63–0.98) and 0.80 (95% CI 0.20–0.96), respectively.

### Statistical analysis

We used the chi-square test, Student’s t-test, and paired t-test to analyze the data. A *p*-value less than 0.05, represented a statistically significant difference between group averages. Statistical analyses were performed using the IBM SPSS Statistics ver. 24.0 (IBM Corp., Armonk, NY, USA).

## Results

Thirty-nine middle fingers of 25 males and 14 females were enrolled. The average age of the 23 healthy volunteers was 32.8 ± 7.7 (range, 27–55) years, and that of the 16 trigger finger patients was 68.6 ± 11.9 (51–86). The Quinnell grading of the patients with trigger finger was as follows: grade 1, one case; grade 2, five cases; grade 3, nine cases; and grade 5, one case. Among the 16 patients with trigger finger, four had diabetes. The descriptive characteristics of the patients with trigger fingers are shown in Table 1.

**Table 1:** Descriptive characteristics of the patients with the trigger finger

	Number of patients
Gender	
Female	10
Male	6
Disease duration (month)[SD]	18.4 [45.2]
Number of affected fingers	
1	4
2	7
3	2
4	3
Diabetes	4

*SD* Standard deviation

The means of each parameter in control cases for pattern A and pattern B are shown in Table 2.

**Table 2:** The means of each parameter in control cases for pattern A and pattern B

	Pattern A (mm)	Pattern B (mm)	*P* value
Parameter a	3.05 [0.15]	3.33 [0.18]	0.02
Parameter b	0.50 [0.04]	0.48 [0.04]	0.63
Parameter c	3.83 [0.14]	3.99 [0.15]	0.26
Parameter d	3.20 [0.19]	3.62 [0.20]	0.004

[ ]: standard deviation

The means of each parameter in patients with a trigger finger for pattern A and pattern B are shown in Table 3.

**Table 3:** The mean value of each parameter in patients with trigger finger for pattern A and pattern B

	Pattern A (mm)	Pattern B (mm)	*P* value
Parameter a	2.78 [0.29]	3.00 [0.32]	0.23
Parameter b	1.45 [0.16]	1.46 [0.16]	0.85
Parameter c	4.59 [0.28]	4.63 [0.35]	0.82
Parameter d	2.74 [0.14]	3.53 [0.15]	< 0.001

[ ]: standard deviation

**Fig. 5:**
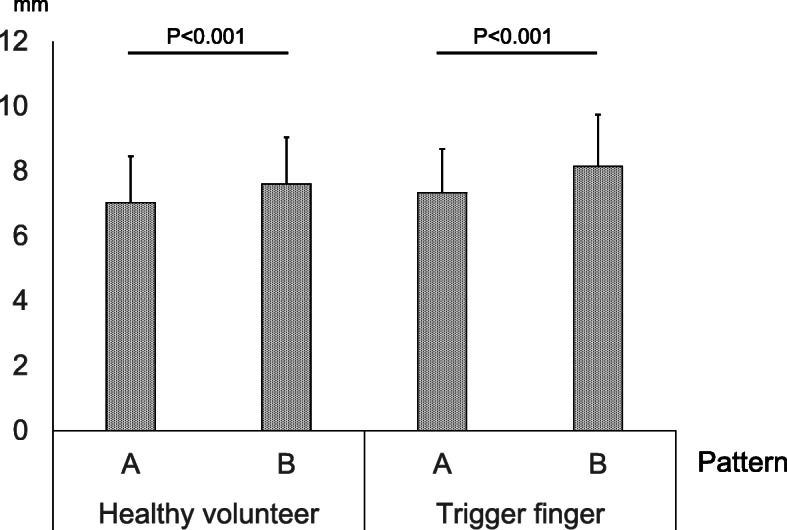
The mean distances of parameter c + d on pattern A and pattern B in each healthy volunteers and patients with trigger finger are shown. Error bars indicate standard deviation

The average diameter of the A1PL (c + d) in healthy volunteers for patterns A and B were 7.03 ± 0.43 mm and 7.64 ± 1.44 mm, respectively, and 7.34 ± 1.35 mm and 8.15 ± 1.59 mm, respectively, in patients with trigger finger. The average diameter of A1PL significantly expanded by 0.57 mm (mean percentage expansion of A1PL: 100 × parameter c + d in pattern B/parameter c + d in pattern A, 8.2%) in healthy volunteers (*P* < 0.001), and 0.81 mm (11%) in patients with trigger finger (*P* < 0.001) (Fig. 5). There was no significant difference between healthy volunteers and patients with trigger finger in terms of the difference in parameter c + d (*P* = 0.141).

We analyzed the relationship between each measurement parameter (a–d) and disease duration, sex, Quinnell grade, number of affected fingers, and the presence or absence of diabetes. Among these variables, only sex was associated with parameter c (*p* = 0.003) and parameter c + d using multivariate analysis (*P* = 0.03, *R*^2^ = 0.40).

## Discussion

In this study, we measured and evaluated the structural changes around the A1 pulley caused by applying an active isometric bending force to a finger flexed at 45° at the MP joint in 23 healthy volunteers and 16 patients with trigger finger using ultrasonography. The average diameters of A1PL significantly increased by 0.57 mm in healthy volunteers and 0.81 mm in patients with trigger finger. This study demonstrated that isometric contraction of the flexor tendon increased the space under the A1 pulley in both healthy volunteers and patients with trigger finger. The mean percentage expansion of A1PL was 8.2% in healthy volunteers and 11% in patients with trigger finger, and while the mean percentage expansion was not very large, these enlargements of the A1PL could improve the stiffness, pain, or catching. A previous study revealed that a strain over 2.0–4.0% resulted in permanent deformation in horse and human tendons [21]. Histological studies on specimens strained to the 1.0, 2.0, 3.0, and 4.0% levels indicate that above the 2.0–3.0% strain level, not all the collagen fibers return to their original unstrained wave pattern. Abrahams et al. also concluded that the stress relaxation phenomenon for the tendon was essentially the same as that observed for other connective tissues.

Miyamoto et al. reported that the stiffness of the A1 pulley in patients with trigger finger was more than that in healthy volunteers [5]. However, in this study, the mean percentage expansion of A1PL was 8.2% in healthy volunteers and 11% in patients with trigger finger. A relatively smaller value of parameter d and a larger tendon diameter in patients with trigger finger in pattern A than that of healthy volunteers could affect the mean percentage expansion of A1PL in both the groups in this study.

To take measurements of all patients easily under the same conditions and increase the reproducibility of this study, we evaluated the expansion of the A1 pulley under isometric contraction of the flexor tendon at the MP joint flexed at 45°. Applying isometric contraction with a more flexed angle of the MP joint may increase the force vector in the direction of expanding the A1PL through the flexor tendon. In clinical practice, it may be more efficient to expand the A1 pulley if the MP joint can be flexed more and the PIP joint can be flexed.

Parameter c of female patients with trigger finger was significantly smaller than that of male patients. These results could be explained by the smaller cross-sectional area of the tendon in females than in males [22, 23].

Parameter a increased significantly upon isometric contraction of the flexor tendon in healthy volunteers. In contrast, there was no difference in it in patients with trigger finger. Even during isometric contraction of the flexor tendon, it could move slightly to the volar side. Volar side shift of the flexor tendon could occur, especially in the distal part of the flexor tendon from the A1 pulley, which could make subcutaneous fat above the A1 pulley slightly redundant and thick. We assumed that parameter a increased owing to the deformation of subcutaneous fat by pressure from the isometrically contracting flexor tendon. Patients with trigger finger have relatively stiffer A1 pulleys; therefore, we assume that parameter a was less affected by isometric contraction of the flexor tendon.

There were no patients with diabetes among the 23 healthy volunteers, and four of the 16 patients with trigger finger were included in this study. Previous estimates of the prevalence of trigger finger in the diabetic population ranged from 5 to 20%, compared with 1–2% in the general population [24]. Patients with diabetes are predisposed to developing trigger fingers compared to the general population. Their symptoms tend to be severe, and multiple fingers are affected bilaterally [24]. However, the use of corticosteroid injections shows a high recurrence rate of trigger finger in diabetic patients [25], while repetitive corticosteroid injections increases the risk of tendon rupture. Moreover, corticosteroid injections can increase blood glucose concentration in patients with diabetes [26]. It may be particularly beneficial for these patients to relieve symptoms by enlarging the A1PL using the A1 pulley stretch.

The limitation of this study is that the stretching effect of the A1 pulley is likely to be enhanced in the MP joint deep flexion and PIP joint flexion positions; however, this was not performed in this study. As mentioned earlier, it is still difficult to obtain an ultrasonic image when the MP joint is deeply flexed, even when using a hockey stick probe. However, we believe that the results of this study are sufficient to understand the effect of MP joint flexion with isometric contraction of the flexor tendon on the expansion of the A1 pulley lumen. Second, it is unclear if the repetitive expansion of th A1PL is retained over a long period. Regardless, tentative expansion of the A1PL appears to have a positive impact on the symptoms of trigger finger. Third, the measurer was not blinded to the group to which the participants belonged to, possibly affecting the results due to experimenter bias.

## Conclusion

We suggest that isometrically applying a flexing force to the finger could expand the A1PL, which is a common site for stenosis of the trigger finger in both healthy volunteers and patients with trigger finger.

## Data Availability

The datasets generated during and/or analyzed during the current study are available from the corresponding author on reasonable request.

## Abbreviations

A1PL: A1 pulley lumen
MP: Metacarpophalangeal
PIP: Proximal interphalangeal
